# Fibrin barriers limit vancomycin penetration into staphylococcal abscess communities and maintain *S. aureus* in an unstressed, transcriptionally-responsive state

**DOI:** 10.64898/2026.01.20.700504

**Published:** 2026-01-20

**Authors:** Parsa Alba Farhang, Timothy Huang, Rezia Era D. Braza, Anjali Anil, Kimberly M. Davis

**Affiliations:** 1 W. Harry Feinstone Department of Molecular Microbiology and Immunology Johns Hopkins Bloomberg School of Public Health, Baltimore, MD, USA

## Abstract

During *Staphylococcus aureus* infection, bacteria are frequently organized into staphylococcal abscess communities (SACs), multicellular bacterial aggregates encased within a host-derived fibrin barrier. Fibrin barriers are thought to insulate SAC-resident bacteria from host immune cells, as strains unable to form these barriers exhibit marked attenuation of virulence in animal infection models. However, the clinical consequences of these structures on antibiotic penetration, and whether the structures also impact bacterial physiology and alter antibiotic susceptibility, remains understudied. Here, we built upon prior work to develop a three-dimensional (3D) collagen gel matrix-based model, utilizing human coagulation components, that supports high throughput *in vitro* SAC formation and is compatible with timelapse microscopy and bulk transcriptional profiling. Using this system, we show that the fibrin barrier protects SACs from clinically relevant dosages of vancomycin by restricting drug penetration. Furthermore, we show that the fibrin barrier maintains stationary phase SACs in an unstressed and transcriptionally responsive state, in stark contrast with stationary phase planktonic *S. aureus* and fibrin barrier-deficient SACs. Together, these findings expand our understanding of SAC fibrin barriers beyond immune evasion to include modulation of antibiotic interactions and bacterial physiological state.

## AUTHOR SUMMARY

Over the course of infection, many bacterial pathogens organize into clustered communities, which limits our ability to fully eliminate bacterial cells with antibiotic treatments. During *Staphylococcus aureus* infection, bacteria form dense clusters surrounded by a protective fibrin barrier derived from host blood clotting machinery, in structures known as staphylococcal abscess communities (SACs). SAC fibrin barriers have been shown to protect against immune cell invasion, but any effects on bacterial physiology and responses to antibiotics remain unclear. In this study, we developed an *in vitro* system to rapidly grow SACs in a 3D collagen gel matrix suspension, closely mimicking human tissue. Using this system, we show that fibrin barriers restrict vancomycin penetration into maturing SACs, protecting them from exposure to this clinically relevant antibiotic. We also show that bacteria within mature SACs are maintained in an unstressed and transcriptionally responsive state when the fibrin barrier is present, in contrast with fibrin barrier deficient SACs. These findings not only suggest that fibrin barriers serve as a physical barrier to drug diffusion but also show the importance of fibrin barriers in bacterial physiology, highlighting their importance in SAC biology and infection persistence.

## INTRODUCTION

*Staphylococcus aureus* is a common causative agent of bacterial infections worldwide, resulting in a high degree of morbidity and mortality (1–5). Effective therapeutic options are scarce due to the rising prevalence of antibiotic resistance, as well as coordinated bacterial morphological structures that make infection clearance challenging (3,6–9). One of these structures is the staphylococcal abscess community (SAC), which is a tight cluster of bacteria encased in a fibrin pseudocapsule and more distal fibrin meshwork, derived from the host coagulation cascade (8,10,11). SACs play an important role in *S. aureus* pathogenesis, as they provide an important physical barrier to immune cells that prevents bacterial clearance. Staphylococci that cannot form SAC-like structures, such as genetic mutants, are markedly attenuated in their virulence in mouse infection models (11–13). Additionally, there is preliminary evidence showing that SACs may protect against certain antibiotics, further highlighting their importance in clinical contexts (14). Expanding our understanding of how these structures form, are maintained, and are disrupted, will provide knowledge that can inform therapeutics aimed at improving clinical outcomes.

Specific virulence factors have been implicated in SAC development and maintenance. The accessory gene regulator (Agr) two-component system (TCS) is a quorum sensing system that controls the expression of a wide array of virulence factors through sensing of auto-inducing peptides (AIPs) released by individual bacteria (15–19). Several Agr-controlled virulence factors are implicated in SAC development, including protein A (*spa*), IsdA adhesin, and staphylokinase (8,12,17,20). Staphylokinase, a secreted protein that converts the clot disrupting enzyme, plasminogen, into its active form, has been shown to disrupt SAC barrier integrity in a small subpopulation of SACs *in vitro*, possibly promoting bacterial dissemination (10,21). It has been shown that Agr is involved in abscess formation in a mouse skin infection model, but Agr is not required for kidney abscess formation (22,23). In addition to the Agr TCS, the *S. aureus* exoprotein (Sae) TCS also plays a key role in SAC biology. The SaeRS TCS, which responds to membrane perturbations, differential nutrient conditions, and oxidative stress, controls several virulence factors directly implicated in SAC formation, with coagulase (*coa*) being the most consequential (17,20,24–27). This TCS has been shown to be necessary for bacterial replication in skin and SAC formation in mouse kidney infection models (23,28,29).

Coa, and a distinct but functionally-related protein von Willebrand factor-binding protein (vWbp), are enzymes that hijack the host coagulation cascade and help encase nascent SACs in their characteristic protective fibrin barrier (26,30). Although it is known that SaeRS promotes *coa* expression, it is not well understood how *vwbp* expression is regulated, and this will not be a focus here (31). Both coagulase and vWbp individually promote blood coagulation through similar and somewhat redundant mechanisms (11,26,30,32). Each enzyme has an N-terminal region that binds and non-proteolytically activates the zymogen form of blood coagulation factor II (prothrombin) and a C-terminal region that binds the blood clotting glycoprotein fibrinogen (11,26,30,32,33). This fibrinogen-prothrombin complex converts soluble fibrinogen into insoluble fibrin that *S. aureus* uses to encase itself in physical fibrin barriers (10,12,26,30,32). Coagulase operates more proximally to the SAC, generating the fibrin pseudocapsule, while vWbp generates fibrin meshwork more distally (10). The resulting SACs provide *S. aureus* with shelter that insulate bacteria from immune cells, making them important for *S. aureus* pathogenesis (8,10,12,14,20,32). Consistent with this, disruption of *coa* or *vwbp* expression or protein function attenuates *S. aureus* virulence *in vivo* (11,12,20,32). However, fundamental questions remain regarding the role of fibrin barriers in SAC biology and pathogenesis in a clinical context. For example, only limited data exists to suggest a protective role of fibrin barriers against antibiotic penetration. It has been shown that gentamicin struggles to penetrate SACs and that exogenously disrupted SACs are more susceptible to gentamicin if treated at 100x the minimum inhibitory concentration (MIC), but assessing antibiotic susceptibility of SACs unable to form endogenous fibrin barriers with a more clinically relevant treatment regimen has yet to be done (14). Additionally, how SACs with and without fibrin barriers differ in development and transcriptional state, remains unknown. Addressing these gaps in knowledge could aid in improving antibiotic treatment outcomes.

Much of the work on SAC formation has been accomplished by infecting mice with mutant *S. aureus* strains and performing endpoint analysis such as viability plating and histology. It is widely speculated that understanding when, in what host niches and in what bacterial subpopulations, the upstream virulence factors (Agr, Sae, Coa) are active is an important next step for understanding SAC biology and ultimately informing therapeutic approaches. Unfortunately, mouse models are largely restricted to endpoint analyses, with technological limitations in tracking individual SACs over space and time. Additionally, findings in a mouse model can sometimes lack translational relevance; *S. aureus* is a human-adapted pathogen and produces virulence factors that have limited or lack activity in mice (34–36). Traditional cell culture-based systems do not bridge this gap because they support growth in two dimensions (2D), failing to capture SAC spherical fibrin-encased morphology.

To address these limitations, we have built upon recent work in other labs to develop an *in vitro* three-dimensional (3D) collagen gel matrix cell culture model to study SACs (10,14,37). This model involves seeding a bacterial-collagen gel matrix suspension into an individual well of a 96-well plate and then overlaying this matrix with media containing essential human coagulation components. Bacteria form fibrin-encased SACs in unique 3D planes, recapitulating key aspects of the structures found in patients during infection. As shown here, host cells, bacterial fluorescent reporter strains, antibiotics, and bacterial mutants can be readily incorporated. Furthermore, each well in this 96-well plate-based system can contain its own matrix with its own unique microenvironment, making it a high throughput system to assess SAC biology. Another novelty of this iteration of the 3D collagen gel system is the ability to perform timelapse microscopy on fluorescent bacterial reporter strains as well as isolate bulk RNA for transcriptional profiling. Together, these features make this model high throughput, translationally relevant, and well positioned to capture nuances in SAC biology at a high temporal resolution.

Here, we will use this model to answer questions regarding the role of fibrin barriers in SAC biology and SAC-antibiotic interactions: (i) whether fibrin barriers are protective against clinically relevant dosages of vancomycin and the potential protection mechanism, and (ii) whether the fibrin barrier impacts the phenotypic state of SAC-resident bacteria and their ability to respond to vancomycin. Our results indicate that fibrin barriers limit antibiotic penetration and also maintain wild-type (WT) mature SACs in an unstressed, transcriptionally responsive, state. In contrast, mutant SACs lacking fibrin barriers exhibit a stressed phenotype prior to antibiotic exposure and lack a transcriptional response to vancomycin. Ultimately, this study further highlights the crucial role of fibrin barriers in SAC antibiotic susceptibility and transcriptional phenotypes.

## RESULTS

### *S. aureus* forms clinically relevant SACs in 3D collagen gel matrix assay

**Figure 1.**
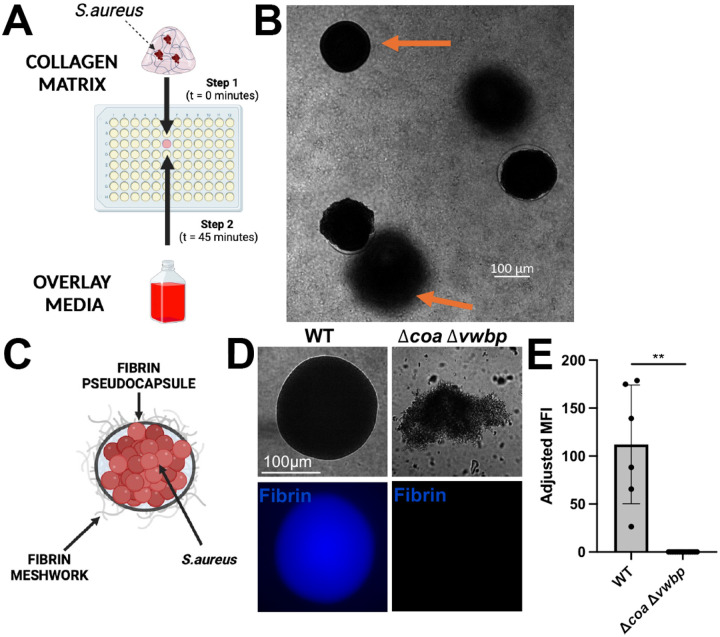
SACs with fibrin barriers form in 3D collagen gel matrix assay in a *coa* and *vwbp* dependent manner. **A)** Schematic of the 3D gel matrix workflow. Log phase *S. aureus* is suspended in a collagen gel and seeded in a well of a 96-well plate. After solidification for 45 min in the 37°C incubator, overlay media (containing human plasma, human fibrinogen, pro-thrombin, and RPMI+L-glutamine) is added. **B)** Representative image taken of the center of a 3D gel matrix containing SACs at 19h of growth. Arrows are highlighting SACs growing in different Z-planes. **C)** Schematic of a staphylococcal abscess community (SAC). **D)** Representative immunofluorescence images of WT and Δ*coa* Δ*vwbp* SACs stained for human fibrin at 18h of growth after media addition. Fibrin was detected using mouse anti-human fibrin antibody and goat anti-mouse antibody (AlexaFluor 350). **E)** Quantification of fibrin staining. Adjusted MFI: total MFI with each strain’s no primary antibody control MFI subtracted (2 biological replicates per strain; 6–14 SACs per strain). Statistics: Welch’s t-test; ** P<0.01.

To generate SACs using the *in vitro* 3D collagen gel matrix assay, log-phase *S. aureus* is suspended in a collagen gel solution then seeded into individual wells of a 96 well plate. After gel solidification, overlay media containing key components of human coagulation biology (plasma, fibrinogen, and pro-thrombin) is layered on top of each collagen gel matrix (Fig 1A). This 3D suspension, coupled with the presence of human coagulation components, supports SAC formation. SACs develop across unique z-planes, which is also observed in mouse infection models (Fig 1B) (23).

Because SACs are defined by their host-derived fibrin structures, we next confirmed the presence of these structures in our system (Fig 1C). To identify the presence of fibrin structures, WT and mutant (Δ*coa* Δ*vwbp*) SACs were grown and probed for the presence of fibrin using immunofluorescence microscopy (38,39). There was a clear presence of fibrin barriers in WT SACs, and not Δ*coa* Δ*vwbp* SACs, which was observed both morphologically (presence and absence of spherical morphology) and through immunofluorescence microscopy (Fig 1D, 1E). Collectively, this data shows that SACs grown in the 3D collagen gel matrix system recapitulate key structural features of those observed *in vivo*.

### SACs display temporal regulation and inter-SAC heterogeneity in virulence gene expression that can be tracked via fluorescence timelapse microscopy

To determine if we can use this system to track SAC gene expression, we incorporated previously described chromosomally-integrated fluorescent reporter strains into the gel matrix system (23). These include *P*_*agrB*_*::gfp*, *P*_*saeP*_*::gfp*, and a GFP^−^ control reporter strains, each in a constitutive mCherry background (*P*_*sarA*_*::mCherry*). The GFP^−^ control reporter strain contains *gfp* inserted without a promoter into the same genomic location to control for possible leaky expression and background autofluorescence.

**Figure 2.**
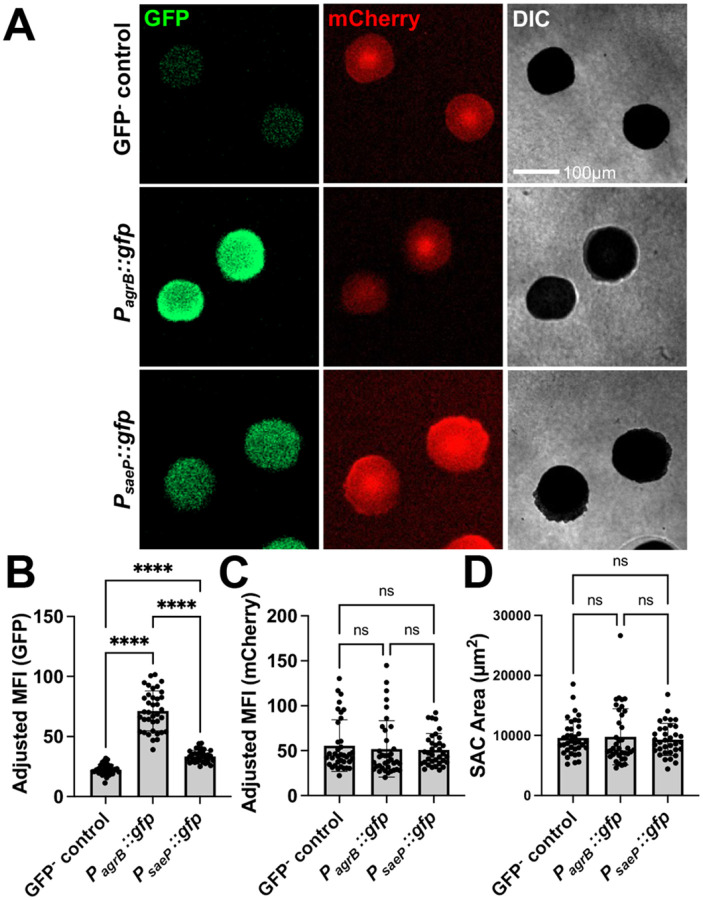
Fluorescent endpoint microscopy detection of Agr and Sae activity in mature SACs in the 3D gel matrix. **A)** Representative images of fluorescent reporter strain SACs grown in the 3D gel matrix 20h after overlay media addition. Each strain contains chromosomally-integrated P_*sarA*_::*mCherry* serving as a constitutive signal. **B-C)** Quantification of SAC fluorescent reporter signal across a population of SACs grown 20h in the 3D gel matrix. Each dot represents the adjusted mean fluorescent intensity (MFI; Total MFI - background matrix signal) of individual SACs (3 biological replicates per strain; 35–37 SACs per strain). **D)** Quantification of SAC area (μm^2^) across a population of SACs grown 20h in the 3D gel matrix. Statistics: **B)** one-way ANOVA and Tukey’s multiple comparisons test; **C & D)** Kruskal Wallace test with Dunn’s multiple comparisons test (at least one of the columns was not normally distributed per Shapiro-Wilk test); **** P<0.0001, ns P>0.05

*P*_*agrB*_*::gfp* expression was significantly heightened in mature SACs (20h of growth), as expected due to the high density of bacteria within each SAC and within a given matrix (Fig 2A, 2B). *P*_*saeP*_*::gfp* showed expression above baseline but below that of *P*_*agrB*_*::gfp*, indicating limited activity in mature SACs (Fig 2A, 2B). mCherry levels and SAC size were consistent across strains, demonstrating that the fluorescent constructs do not alter SAC biology (Fig 2A, 2C, 2D).

**Figure 3.**
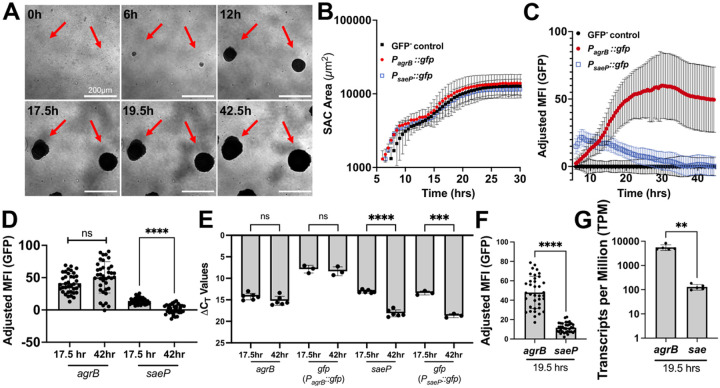
Fluorescent timelapse microscopy shows Sae activity decreases while Agr activity increases in maturing SACs in the gel matrix. **A)** Representative images of SACs grown in 3D gel matrix overtime. Time represents time elapsed after overlay media addition. **B)** Average SAC area (μm^2^) per strain per timepoint in 30 min increments (3 biological replicates per strain; 31–38 SACs per strain). **C)** GFP adjusted mean fluorescent intensity (MFI) of fluorescent reporter strain SACs in 30 min increments (3 biological replicates per strain; 31–38 SACs per strain). Average GFP^−^ control GFP signal per timepoint was subtracted from the GFP MFI of the *agrB* and *saeP* reporter strains. **D)** GFP adjusted MFI of individual SACs for each strain at designated timepoints (3 biological replicates per strain; 35–38 SACs per strain). **E)** The centers of individual gel matrices were extracted using a biopsy punch, then processed for RT-qPCR. ΔC_T_ values were generated by subtracting 16S rRNA C_T_ from the target gene C_T_. Each point represents one matrix (6 biological replicates; 3 P_*agrB*_::*gfp*, 3 P_*saeP*_::*gfp*). **F)** GFP adjusted MFI of individual SACs for each strain at 19.5h of growth (3 biological replicates per strain; 35–37 SACs). **G)** Transcripts per million (TPM) from bulk RNA sequencing of whole matrices after 19.5h of growth (5 biological replicates; non-fluorescent WT strain). Statistics: **D-F**) Welch’s t-tests for each pairwise comparison with a Bonferroni multiple comparisons correction to adjust each p value where applicable; **G)** Mann Whitney U test was used due to a non-normal distribution according to a Shapiro-Wilk test; **** P<0.0001, **P<0.01, ns P>0.05.

A specific limitation of studying SAC biology within mouse models of infection is that analysis is typically restricted to endpoints, preventing the tracking of individual SACs during maturation. SACs within infected mouse tissue are also seeded asynchronously, making it difficult to determine whether SACs represent similar developmental stages at a given endpoint (23). In contrast, our gel matrix system enables the use of automated timelapse microscopy to follow SAC development continuously, both across wells and within individual matrices (Fig 3A). Target gene expression can also be continuously assessed using fluorescent reporter strains in the matrix system. Using 30 minute automated imaging intervals, we observed that *P*_*saeP*_::*gfp, P*_*agrB*_::*gfp*, and GFP^−^ control strains grew exponentially and then gradually plateau after ~10–15h as SACs fully mature and enter stationary phase (Fig 3B). Distinct gene expression patterns were observed based on GFP signal (Adjusted MFI), as *P*_*saeP*_::*gfp* expression peaked early in SAC development and gradually declined as SACs mature, whereas *P*_*agrB*_::*gfp* expression gradually increased overtime before plateauing in the stationary phase of SAC growth (Fig 3C, D). These trends were confirmed via RT-qPCR (ΔC_T_ values) of bulk RNA isolated from gel matrices (Fig 3E). Furthermore, *P*_*agrB*_::*gfp* fluorescent signal is higher than that of *P*_*saeP*_::*gfp* in mature SACs (Fig 3F), which was corroborated using bulk RNA-seq on matrix-derived RNA at 19.5 hrs (Fig 3G). Together, these data demonstrate that microscopy of fluorescent reporter SACs in the gel matrix system provides accurate readouts of gene target transcriptional activity overtime.

**Figure 4.**
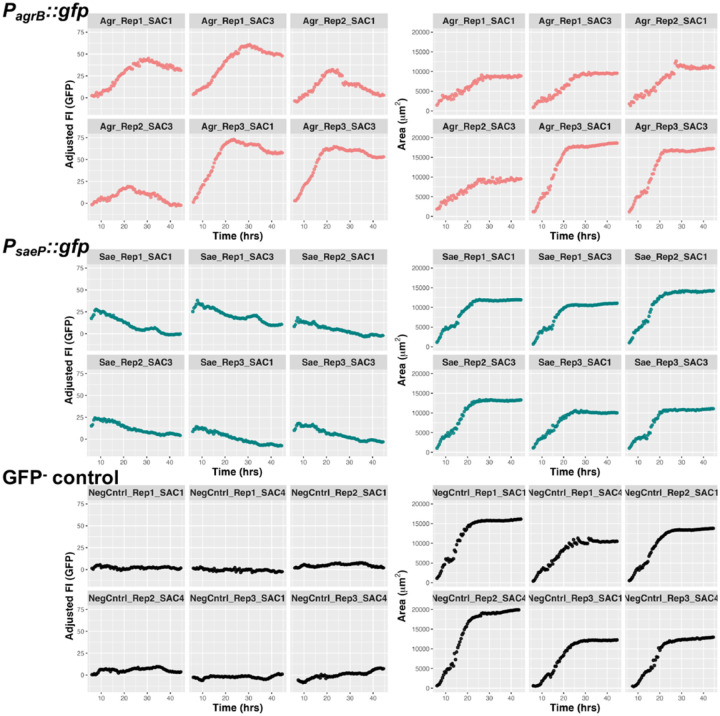
SACs grown in gel matrix display interSAC heterogeneity in growth and virulence factor expression. Area (μm^2^) and adjusted GFP fluorescent intensity (FI) of individual SACs from experiments in Fig 3B–C. SACs are identified in the order of reporter strain (i.e. *P*_*agrB*_*::gfp* is denoted by “Agr”), biological replicate, and the individual SAC number within that biological replicate and strain.

In addition to measuring a SAC population throughout a well, we are able to segregate the size and fluorescent intensity readouts of each individual SAC (Fig 4). These single-SAC analyses highlight that SACs grown in this system display inter-SAC heterogeneity in size and transcriptional activity, similar to the heterogeneity that has been observed during mouse infection (23).

While not a focus of this manuscript, this gel matrix model can also be used to assay host-pathogen interactions, in line with previous iterations of this assay (10,14,37). Primary human neutrophils (PMNs) were co-cultured with nascent SACs and assessed for changes in SAC dynamics (**Fig S1A**). While colony forming units (CFUs) were unchanged **(Fig S1B**), SAC size was restricted (**Fig S1C**), and *P*_*saeP*_::*gfp* expression was heightened (**Fig S1D**) in SACs co-cultured with PMNs. These results provide compelling evidence for assaying host-pathogen interactions in this system and offer another avenue for future investigation.

### Fibrin barrier is protective against vancomycin in log-phase SACs

Given that fibrin barriers form in this system in a *coa* and *vwbp*-dependent manner (Fig 1D), we specifically sought to determine whether the presence of the fibrin barrier could impact vancomycin treatment efficacy. Prior mouse infection studies have shown that fibrin barriers protect SAC residing bacteria, as mutants lacking these structures struggle to establish infection (8,20). Because of the attenuation of these strains (Δ*coa* Δ*vwbp*), it has been difficult to assess the specific role of the fibrin barrier during infection and antibiotic treatment. While the exact mechanism of protection has remained elusive, some evidence suggests that the fibrin barriers could restrict drug penetration into fully mature SACs (14).

To test the role of the fibrin barrier in the antibiotic susceptibility of log phase SACs, WT, Δ*coa Δvwbp*, and Δ*sae* SACs were grown into late log phase and treated with a clinically relevant dosage of 50μg/mL vancomycin, the frontline drug used to treat MRSA, for 5h (40–42). The Δ*sae* strain was used here as a control since *coa* is expressed downstream of SaeRS activation (31). While all three strains grow similarly in culture, the Δ*sae* strain grows slightly faster than the others in the gel matrix (**Fig S2A-C**). To account for this difference in growth dynamics, the WT and Δ*coa Δvwbp* SACs were treated with vancomycin after 8.5h of growth and the Δ*sae* after only 6.5h, ensuring they were treated at analogous late-log phase growth stages.

**Figure 5.**
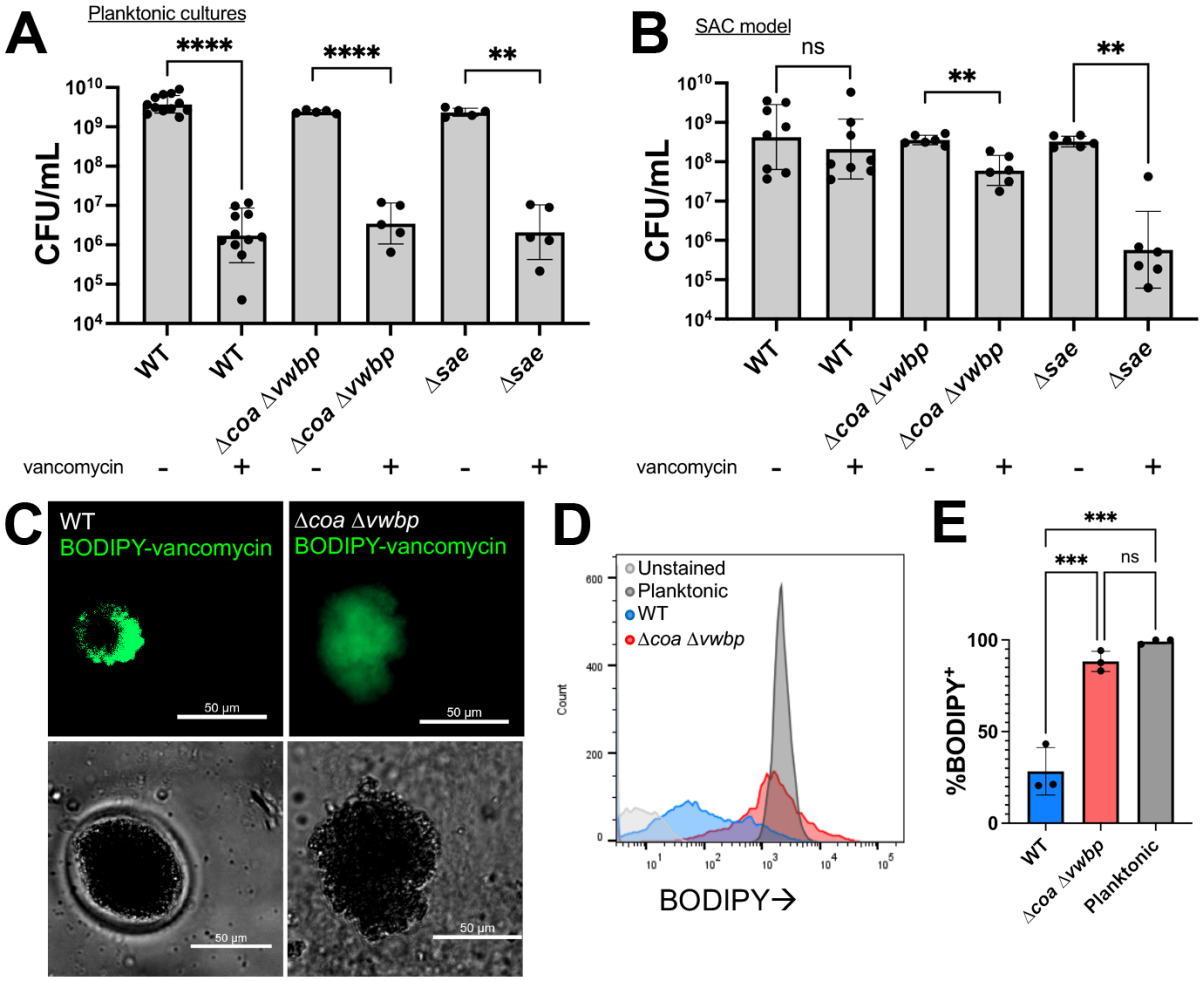
Fibrin barrier protects log-phase SACs from vancomycin. **A)** Vancomycin susceptibility of log-phase planktonic cultures. **B)** Vancomycin susceptibility of log-phase SACs. All strains and conditions were treated with 50μg/mL vancomycin for 5h. WT and Δ*coa* Δ*vwbp* SACs were treated at 8.5h of growth, while the faster growing Δ*sae* strain was treated after 6.5h of growth (Fig S2). **C)** Representative images of SACs grown 8.5h in a glass chamber slide to improve imaging resolution. Matrix reagents were scaled appropriately to account for the larger surface area of each well. SACs were stained with 4μg/mL BODIPY-vancomycin. **D)** Representative flow cytometry histograms of each condition after 8.5h of growth stained with BODIPY-vancomycin (unstained control: WT planktonic cells). **E)** Percentage of BODIPY^+^ bacteria for WT and Δ*coa* Δ*vwbp* SACs as well as WT planktonic bacteria (3 biological replicates per condition). Statistics: **A-B)** Welch’s t-test or Mann Whitney U test depending on the normality of each column in each pairwise comparison as determined by Shapiro-Wilk test. Multiple comparisons were accounted for using a Bonferroni correction; **E)** one-way ANOVA with Tukey’s multiple comparisons test; ****P<0.0001; *** P<0.001; **P<0.01; ns P>0.05.

In log phase planktonic culture, all three strains displayed similar levels of vancomycin susceptibility (Fig 5A). In contrast, log-phase WT SACs lacked susceptibility, with no change in CFUs upon vancomycin addition relative to untreated controls, whereas Δ*coa* Δ*vwbp* and Δ*sae* mutant SACs saw significant CFU reductions (Fig 5B). To determine if WT SAC tolerance was due to the fibrin barrier restricting vancomycin penetration, WT and Δ*coa Δvwbp* SACs were stained with the fluorescent BODIPY-vancomycin conjugate after 8.5h of growth, alongside unstained and stained planktonic control cells. Fluorescent microscopy and flow cytometry data indicate that the fibrin barrier restricts vancomycin diffusion into WT SACs, while Δ*coa Δvwbp* SACs were uniformly penetrated, similar to planktonic cells (Fig 5C, 5D, 5E). These results indicate that the fibrin barrier protects actively growing SACs from vancomycin by serving as a diffusion barrier.

### Stationary phase SACs are transcriptionally responsive to vancomycin

**Figure 6.**
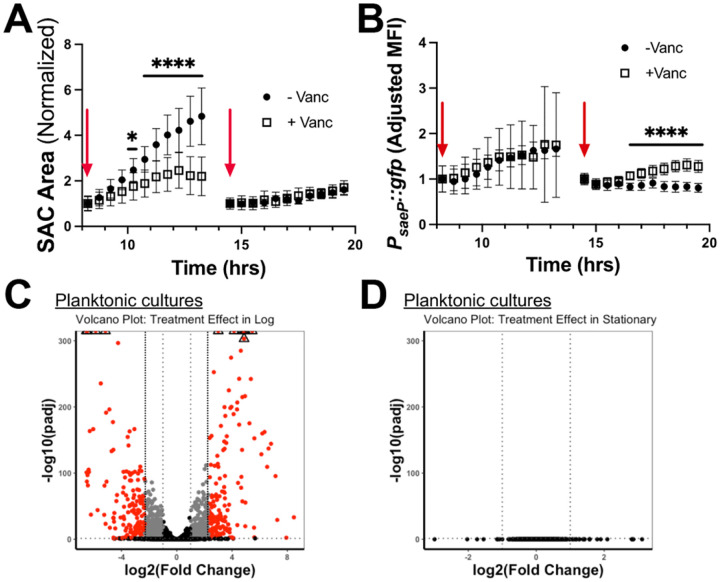
Stationary phase WT SACs respond transcriptionally to vancomycin. **A-B)** *P*_*saeP*_*::gfp* SACs were grown in the gel matrix system for 8.25h (log) or 14.5h (stationary) in triplicate. 0 or 50μg/mL of vancomycin was added in technical replicates of each biological replicate, then imaged overtime at 37°C. SAC size (μm^2^) and adjusted mean fluorescent intensity (MFI) were tracked (3 biological replicates; 13–24 SACs per timepoint). **C)** WT *S. aureus* overnight cultures were back-diluted 1:100 in TSB (3 biological replicates) and grown for 2h on the 37°C rotor. Technical replicates per biological replicate were then treated with 0 or 25 μg/mL vancomycin for 2h on a 37°C rotor, fixed, and processed for RNA isolation. Bulk RNA-seq analysis revealed 296 differentially regulated genes (|log_2_FC| >2.32 and Padj <0.05) when comparing untreated and treated groups. In the volcano plots, black dots represent genes with Padj >0.05 and/or |log_2_(Fold Change)| <1; grey dots indicate genes with Padj < 0.05 and |log_2_(Fold Change)| >1 but < 2.32; red dots indicated genes with Padj < 0.05 and |log_2_(Fold Change)| >2.32. **D)** The same experiment was performed with *S. aureus* grown to stationary phase (vancomycin was added after 5.5h of growth). Bulk RNA-seq analysis showed 0 differentially regulated genes when comparing untreated and treated groups. Statistics: **A-B)** multiple unpaired t-tests and multiple comparisons were corrected for using the Holms-Sidak method; **** P<0.0001; * P<0.05.

After observing that fibrin barriers protect log phase SACs from vancomycin, we were interested in understanding if stationary phase SACs were similarly protected from vancomycin, and if bacterial transcriptional changes could impact survival during exposure. Log (8.25h) and stationary (14.5h) phase WT SACs were challenged with vancomycin (Fig 6A, 6B). To assess the presence of a transcriptional response we used the *P*_*saeP*_::*gfp* reporter strain, as vancomycin has been shown to activate SaeRS in *S. aureus* (43,44), and timelapse microscopy was used to capture transient transcriptional changes. Despite a clear restriction of log phase SAC area in vancomycin-containing matrices, there was no differential expression of *saeP* when compared to untreated conditions (Fig 6A, 6B). In contrast, while stationary phase SACs with and without vancomycin displayed no difference in SAC size, SACs in vancomycin-containing matrices had an increase in relative *saeP* expression. This was surprising given the conventional wisdom that stationary phase bacteria tend to be less metabolically active and, therefore, less responsive to drugs than their log phase counterparts (45–47).

The presence of a differential transcriptional response in stationary phase SACs was noteworthy, and to determine if this was unique to SACs compared to planktonic growth, we treated WT log and stationary phase planktonic *S. aureus* with vancomycin (25μg/mL). This lower concentration (vs. 50μg/mL for SACs) was used to prevent excess cell lysis, and subsequent controls confirmed that 25μg/mL and 50μg/mL of vancomycin produced similar viability and transcriptional outcomes in planktonic cells (**Fig S3A, S3B**). We then assessed the global impact of vancomycin treatment (25μg/mL for 2h) on planktonic cells via bulk RNA-seq and observed a robust differential expression of genes in log phase but no significant changes in stationary phase (Fig 6C, 6D). Several of the genes differentially regulated upon treatment in log-phase align with previously reported findings. For example, *vraR* and *vraX*, part of the *vra* family of genes that are highly responsive to vancomycin and play a role in toxic compound export and the cell wall stress response, were both upregulated upon vancomycin exposure (**Supplemental Table 1**) (44,48). This data indicates that stationary phase SACs are transcriptionally responsive to vancomycin, whereas stationary phase planktonic *S. aureus* are not, suggesting something about the SAC morphology allows bacteria to respond to vancomycin.

### WT SACs respond to vancomycin, while phase-matched Δ*coa* Δ*vwbp* SACs exhibit constitutive stress and are unresponsive

After observing that stationary phase WT SACs mount a transcriptional response to vancomycin, we next asked whether this transcriptional responsiveness was a fibrin barrier-dependent phenomenon. To test this, WT and Δ*coa* Δ*vwbp* SACs were grown into stationary phase, treated with vancomycin or left untreated, and RNA was isolated for bulk RNA sequencing.

**Figure 7.**
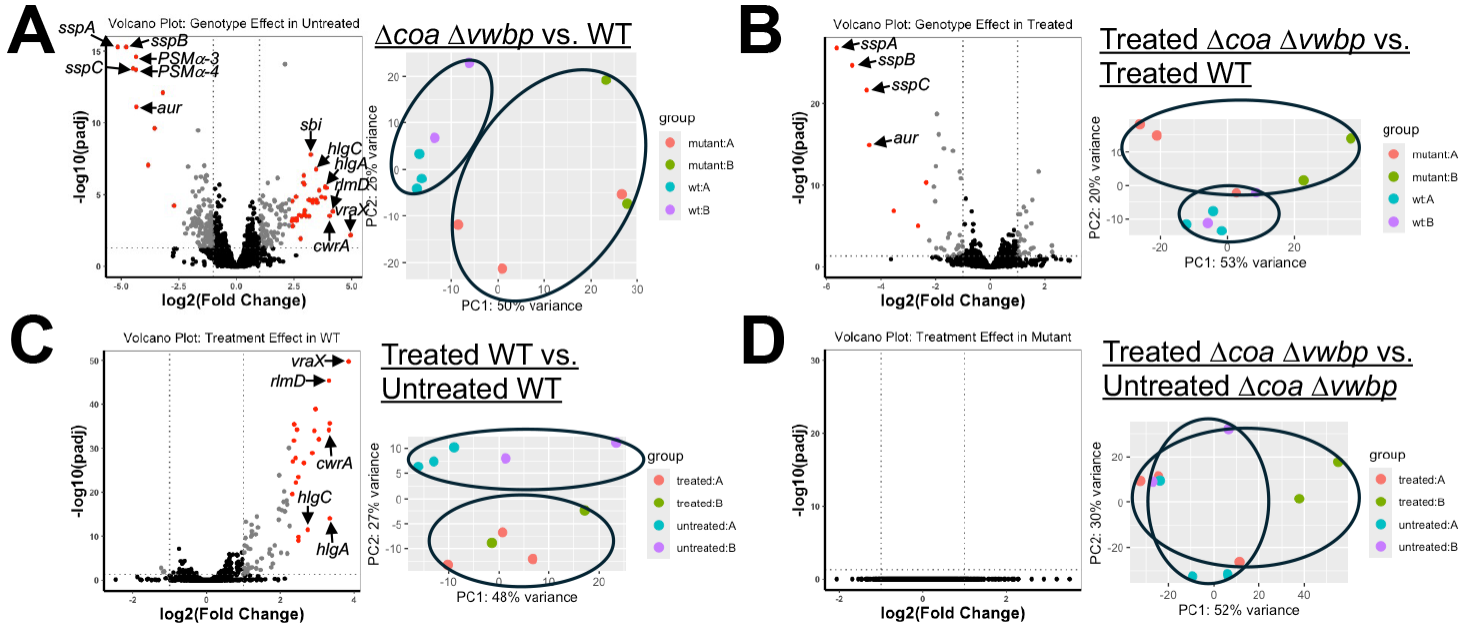
Stationary phase WT SACs respond transcriptionally to vancomycin, whereas Δ*coa* Δ*vwbp* SACs remain in a pre-stressed, unresponsive state. **A-D)** Differential gene expression analysis of indicated RNA-seq comparisons. Left panels: volcano plots showing genes and their -log_10_(Padj) v. log_2_(Fold Change). Black dots represent genes with Padj >0.05 and/or |log_2_(Fold Change)| <1; grey dots indicate genes with Padj < 0.05 and |log_2_(Fold Change)| >1 but < 2.32 (between 2–5 fold); red dots indicated genes with Padj < 0.05 and |log_2_(Fold Change)| >2.32 (≥5 fold). Annotated labels highlight selected genes of interest. Right panels: PCA plots showing separation of comparison groups, with samples colored based on comparison group (ex. treated or untreated) and batch. Ellipses outline comparison groups.

Comparison of untreated WT and Δ*coa*Δ*vwbp* SACs revealed clear transcriptional differences, as the groups segregated upon PCA analysis (Fig 7A). When comparing differentially expressed genes, there were clear signatures of a stress response in Δ*coa* Δ*vwbp* SACs. Canonical stress response genes, such as *msrA* and *msrB* were significantly upregulated, while an array of other genes thought to play a role in *S. aureus* stress response were also elevated (Table 1, **Supplemental Table 2**). These include the *vra* genes (*vraX, vraR, vraS, vraD, vraE*); *hlgA* and *hlgC*, pore-forming toxins that combat immune cells (leukocidins); *cwrA*, a cell wall inhibition responsive protein that increases cell wall integrity; *rlmD*, a 23S rRNA uridine methyltransferase known to impact antibiotic interaction with ribosomes; and *sbi*, which binds immunoglobulin to inhibit humoral and complement-mediated immunity (44,48–53). GO-enrichment analysis also suggests a heightened stress response in Δ*coa* Δ*vwbp* SACs, as well as increased protein turnover (**Supplemental Table 3**). In contrast, WT SACs expressed V8 protease genes (*sspA, sspB, sspC, aur*) and several phenol-soluble modulin genes (PSMα 2–4), which could together promote dissemination and immunomodulation (17,54,55). A comparison between vancomycin treated Δ*coa* Δ*vwbp* and treated WT SACs similarly highlighted that V8 protease gene (*sspA, sspB, sspC, aur*) expression was heightened in treated WT SACs (Fig 7B) (Table 1). However, the treated SACs lacked clear segregation by PCA analysis (Fig 7B), also suggesting some similarities in their transcriptional state after vancomycin exposure.

To determine the impact of treatment on WT SACs, we compared treated and untreated samples, and saw clear separation via PCA analysis, indicating distinct transcriptomes (Fig 7C). Interestingly, the differentially expressed genes were largely upregulated, and many of these were also significantly expressed in untreated Δ*coa* Δ*vwbp* SACs when compared to untreated WT (Fig 7A) (such as *vra* genes, *cwrA*, *hlgA, hlgC, cwrA*, and *rlmD*) (Table 1, **Supplemental Table 2**). Several of these genes, including *vraR, cwrA*, and *rlmD*, were also upregulated in log-phase planktonic bacteria treated with vancomycin relative to untreated bacteria, underscoring similarities in responses to the same stressor (Fig 6C). However, it is important to note there were many differentially expressed genes unique to each data set (**Supplemental Table 1, Supplemental Table 2**). To facilitate comparison with fluorescent reporter data in Fig 6B, which assessed stationary phase *P*_*saeP*_*::sGFP* SACs treated with vancomycin, we examined expression of *saeR* and *saeS* in the RNA-seq dataset. Both genes were significantly upregulated in vancomycin treated WT SACs relative to untreated, with log_2_ fold changes of 1.17 (*saeR*) and 1.12 (*saeS*) and adjusted p-values of 0.0026 and 0.00027 respectively. These transcriptional changes are consistent with the fluorescent readout from Fig 6. Consistent with our expectations going into the experiment, the sequencing data indicated an upregulation in stress response-associated genes during vancomycin exposure. This was consistent with GO term analyses, although the most enriched term, ‘stress response’ did not reach statistical significance (P=0.057) (**Supplemental Table 3**).

We noted significant similarities in the genes expressed by WT SACs in response to vancomycin and the genes expressed by Δ*coa* Δ*vwbp* under untreated conditions, both of which pointed to cell envelope stress response genes. These data indicated that Δ*coa* Δ*vwbp* SACs expressed a ‘stressed’ signature prior to treatment, and we then asked whether vancomycin altered this response by comparing untreated and treated Δ*coa* Δ*vwbp* SACs. We found there was no segregation of treatment groups by PCA analysis and 0 differentially expressed genes, suggesting that Δ*coa* Δ*vwbp* SACs lacked a transcriptional change in response to vancomycin (Fig 7D). These findings indicated that mature SACs lacking a fibrin barrier exist in a stressed transcriptional state, whereas WT SACs upregulate an analogous stress response when vancomycin exposure occurs. Given these experiments were completed in the absence of infection bottlenecks and heterogenous encounters with immune cell populations, these findings demonstrate a more nuanced role for SAC fibrin barriers than previously reported. Fibrin barriers seem to not simply provide a diffusion barrier that insulates SAC-dwelling bacteria from host cells and antimicrobials, but also maintain encased bacteria in a phenotypically distinct, responsive, state.

### No difference in vancomycin susceptibility, regrowth potential, and PMN cytotoxicity between stationary phase WT and Δ*coa* Δ*vwbp* SACs

**Figure 8.**
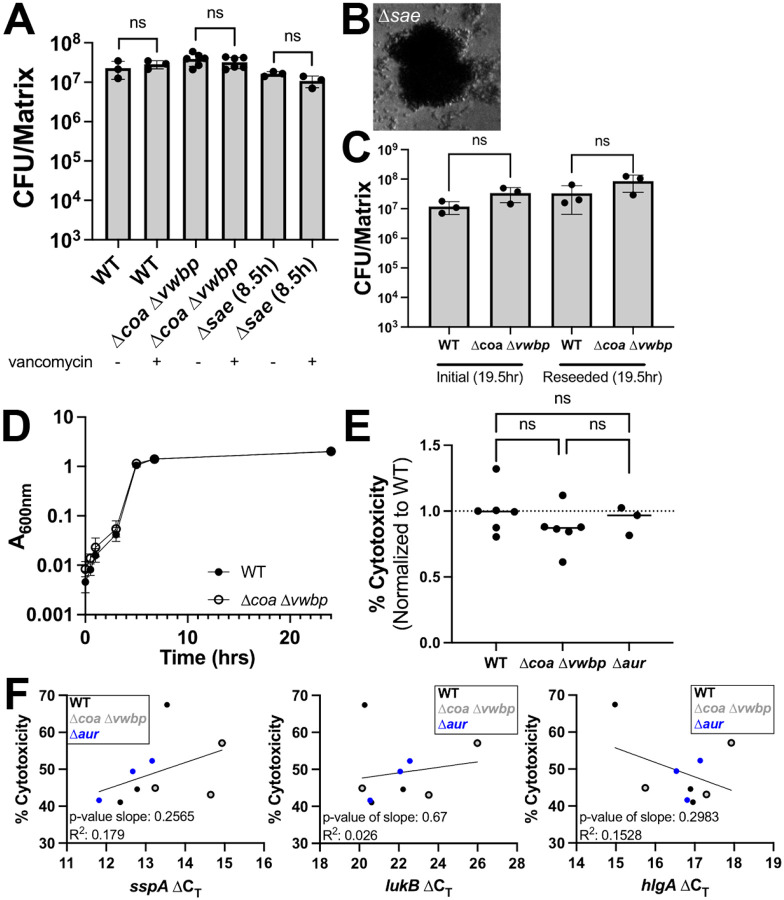
Stationary phase WT and Δ*coa* Δ*vwbp* SACs show no differences in vancomycin susceptibility, regrowth potential, or neutrophil cytotoxicity. **A)** Viability of stationary phase WT, Δ*coa* Δ*vwbp*, and Δ*sae* SACs treated with 0 or 50 μg/mL vancomycin for 5h. All three strains displayed vancomycin tolerance (3–6 biological replicates per strain). **B)** Representative image of mature Δ*sae* SAC. **C)** Reseeding assay. Stationary phase SACs were grown for 19.5h, digested and assessed for CFUs, and reseeded into new matrices. After 19.5h of growth, matrices were again digested and assessed for CFUs (3 biological replicates per strain). **D)** Subset of digested SACs after second 19.5h growth were inoculated into TSB, and A_600_ was tracked overtime (3 biological replicates per timepoint per strain). **E)** Cytotoxicity of PMNs exposed to SAC culture supernatants from WT, Δ*coa* Δ*vwbp*, and Δ*aur* strains, measured by LDH release. Experiment was repeated across two batches, and % cytotoxicity values were normalized to average value of PMNs cultured with WT SAC supernatant within the same batch (3–6 biological replicates per strain). **F)** Simple linear regression comparing toxin transcript levels (ΔC_T_) with corresponding unnormalized PMN % killing (3 biological replicates per strain had both cytotoxicity readout and ΔC_T_ values). Strains are denoted by symbols in panel legend. Statistics: **A)** Welch’s t-test with Bonferroni correction for multiple comparisons; **C)** Welch’s t-tests with Bonferroni correction for multiple comparisons; **E)** one-way ANOVA and Tukey’s multiple comparisons test; ns P > 0.05.

To understand whether the transcriptional differences observed between stationary phase WT and Δ*coa* Δ*vwbp* SACs translated into functional differences, we first assessed the impact on vancomycin susceptibility. Stationary phase WT, Δ*coa* Δ*vwbp*, and Δ*sae* SACs were treated with 50μg/mL vancomycin for 5h and compared to untreated controls. The Δ*sae* strain was used again as a control since *coa* is expressed downstream of SaeRS activation, and also because a subset of genes in the RNA-seq dataset are expressed downstream of SaeRS (i.e. *hlgA, hlgC*). In contrast with exponential phase results, all three strains lacked vancomycin sensitivity in stationary phase (Fig 8A). We also confirmed the Δ*sae* strain lacked the fibrin barrier (Fig 8B).

We hypothesized that the stressed phenotype observed in Δ*coa* Δ*vwbp* SACs could impact the ability for Δ*coa* Δ*vwbp* to resume growth in the context of infection, following dissemination and re-seeding of new infection sites. To test this hypothesis, we grew WT and Δ*coa* Δ*vwbp* mutant SACs into stationary phase then reseeded them in new matrices and measured viable counts after 19.5h. There were no differences in CFUs between strains, indicating the Δ*coa* Δ*vwbp* transcriptional signature did not come at a fitness cost in its ability to form new mature SACs (Fig 8C). In addition to measuring viable counts within SACs, we also inoculated TSB with these WT and Δ*coa* Δ*vwbp* reseeded bacteria and assessed growth in liquid culture. We saw no difference in growth between strains via optical density, indicating that stationary phase Δ*coa* Δ*vwbp* SACs were able to resume growth with kinetics similar to the WT strain.

In the RNA-sequencing data, we saw a significant increase in expression of several toxins that can target human neutrophils (PMNs) (e.g. *hlgAB, hlgCB, lukAB*) in untreated Δ*coa* Δ*vwbp* SACs relative to untreated WT SACs (see full supplemental RNA-seq datasets for *lukAB*; [log_2_ fold change] above 1 but less than 2.32 cutoff) (17). We hypothesized that Δ*coa* Δ*vwbp* SACs may release heightened levels of toxins into the culture supernatant compared to WT, and that this would result in heightened human PMN cytotoxicity. PMNs were isolated from human blood and exposed to culture supernatant from WT or Δ*coa* Δ*vwbp* SACs, and cytotoxicity was assessed by PMN LDH release relative to the WT strain. Surprisingly, WT samples initially appeared to promote more cytotoxicity than Δ*coa* Δ*vwbp*, and an aureolysin mutant (Δ*aur*) was also included to determine if V8 protease activity could contribute to any observed effects (56). However, we did not observe significant differences amongst the groups (Fig 8E). To confirm that SACs exhibited the expected transcriptional signatures, RT-qPCR was also performed with a subset of biological replicates. Some genes, such as *hlgA* and *lukB* (Fig 8F, middle and right panels), did not display a consistent upregulation in Δ*coa* Δ*vwbp* SACs across all biological replicates, in contrast with patterns observed in the RNA-seq data from Figure 7. Other targets, like *vraR* and *sspA*, showed consistent regulation across all biological replicates, directionally aligning with the RNA-seq data (Fig 8F, left panel: *sspA*). These discrepancies may reflect differences in pooled human plasma sources between experiments, as supply chain constraints necessitated switching vendors. Finally, to understand if there was a correlation between levels of specific transcripts and cytotoxicity, we performed linear regression analyses (Fig 8F). Here, lower ΔC_T_ values indicate higher transcript levels, and if heightened transcript levels are linked to increased cytotoxicity, you would expect a negative correlation. We observed this trend with *hlgA*, but this correlation was not significant (Fig 8F, right panel).

In all, while there is compelling evidence showing the fibrin barrier substantially alters the transcriptional state of stationary phase SACs, these differences did not correspond with measurable changes in vancomycin susceptibility, regrowth potential, or PMN cytotoxicity. The functional significance of this observed fibrin-dependent transcriptional state remains an open question.

## DISCUSSION

Here we expanded upon prior work to develop a refined 3D collagen gel matrix model for studying staphylococcal abscess community (SAC) biology. This iteration offers several novel advantages: i) it utilizes a 96-well plate for high-throughput analyses, ii) supports time-lapse microscopy, and iii) enables recovery of total RNA for bulk transcriptomic analyses. Using this system, we observe that *agr* is expressed as SACs mature, whereas *sae* is expressed early and declines over time. *agr* expression likely increases due to an accumulation of AIP in the local environment, while early Sae activity may be necessary to drive *coa* expression for proper fibrin pseudocapsule and MAM formation as SACs mature. To our knowledge, this represents the first high temporal resolution monitoring of these key virulence factors over the course of SAC development.

We were interested in utilizing this system to understand fibrin barrier function, and its possible role during antibiotic treatment, with a focus on vancomycin due to its clinical relevance. Prior investigation with a similar model assessed SAC-gentamicin interaction, and provided representative images that indicated fibrin barriers restricted diffusion of fluorescently-labeled gentamicin (14). Moreover, mature SACs displayed no susceptibility to gentamicin, unlike isogenic planktonic *S. aureus* treated in parallel. Plasmin pretreatment was used to implicate fibrin barriers, however two limitations complicated interpretation: i) SACs were in stationary phase and therefore less likely to display sensitivity to gentamicin, and ii) plasmin would likely only degrade the outer edges of the fibrin structure and may not completely ablate barrier function (14). To address these limitations, we used *S. aureus* strains with clean deletions of *coa* and *vwbp* and treated SACs with a clinically relevant dosage of vancomycin during active growth. In line with this previous study, we also observed restriction of fluorescently-labeled antibiotic to the periphery of WT SACs. However, as we probed SAC antibiotic sensitivity at different growth phases, we found the fibrin barrier protected SACs from antibiotic-mediated killing throughout SAC maturation. In contrast, fibrin-deficient mutant SACs saw a complete diffusion of vancomycin and a significant drop in CFUs in log phase compared to untreated controls. These findings together provide compelling evidence that fibrin barriers protect SAC-resident bacteria from antibiotics by restricting drug diffusion.

To further probe the role of the fibrin barrier, we compared the transcriptional responses of SACs to vancomycin. Surprisingly, stationary phase SACs were transcriptionally responsive, in contrast to stationary phase planktonic culture, which showed no response to vancomycin (Fig 6B, 6D). This compelled us to perform RNA-seq on stationary phase WT and Δ*coa* Δ*vwbp* SACs with and without vancomycin, the first such transcriptional profiling of SACs to our knowledge. WT SACs were indeed transcriptionally responsive to vancomycin but SACs lacking a fibrin barrier were completely nonresponsive, exhibiting a stressed signature prior to and following treatment. Given these SACs were grown in the absence of infection bottlenecks and other stressors associated with a heterogenous host environment, these results could be attributed to the absence of the fibrin barrier. Thus, in addition to protecting growing SACs from vancomycin, fibrin barriers may aid in maintaining mature SACs in a relatively unstressed and transcriptionally-responsive state. With the exception of an *in vitro* study of *M. tuberculosis* granulomas, ours is one of the only studies to directly dissect the roles of individual structural components within bacterial aggregates on the phenotypes of resident bacteria (57).

Although the phenotypic differences between WT and fibrin-deficient SACs are clear, the underpinning mechanism remains unresolved. The fibrin barrier may physically interface with bacterial surface receptors, influencing intracellular signaling pathways akin to cells interfacing with extracellular matrix components. Additionally, given the fibrin barrier can restrict vancomycin diffusion, these structures could also restrict nutrient access or, conversely, be better at retaining them within the SAC structure. Nutrients, such branched chain amino acids (BCAA), modulate the expression of key virulence factors in planktonic *S. aureus* culture (27). For example, BCAAs have been shown to inhibit *sae* expression. Our RNA-seq data showed modest *sae* expression in Δ*coa* Δ*vwbp* SACs (relative to WT SACs), which could be explained by an inability to retain nutrients like BCAAs within the SAC structure due to a lack of fibrin barriers. The fibrin barriers may also sequester bacterial-derived byproducts, such as AIP, impacting Agr and downstream signaling pathways.

In addition to the mechanistic underpinnings of this fibrin-dependent SAC phenotype, it is also of interest to understand the possible translational importance of this phenomenon. Mature SACs without fibrin barriers were not susceptible to vancomycin (unlike their log phase counterparts), but resumed active growth as readily as WT SACs, and displayed no increase in cytotoxicity to PMNs despite expression of neutrophil-targeting toxin transcripts. Proteomic analysis could help clarify if transcriptional differences are manifesting at the protein level. Heterogeneity in responses may also impact these cytotoxicity results, as the SAC transcriptional changes were not as pronounced in this subset of experiments. Single-cell or small population RNA-seq could also capture intraSAC heterogeneity more fully. Further experimentation of WT and fibrin-deficient SACs in the presence and absence of host factors such as neutrophil co-culture or altered nutritional conditions could better mimic specific tissue niches and may aid in developing translational applications. Together, these would deepen our understanding of how fibrin barriers contribute to SAC development and pathogenesis.

## MATERIALS & METHODS

### Bacterial Strains & Growth Conditions

The *S. aureus* WT USA300 LAC isolate was used as the parent background for all experiments in this study, including reporter and deletion strains. These strains have been previously described (23,38,39,42,56). *S. aureus* was grown on tryptic soy agar (TSA) plates at 37°C to isolate single colonies. Overnight cultures were inoculated with a single bacterial colony into tryptic soy broth (TSB) and incubated at 37°C with rotation for 13–20 hrs. Back-dilutions were made in fresh TSB and were placed again on the 37°C rotor for the indicated time. Fluorescent reporter strains contain a kanamycin resistance cassette and were cultured with 50μg/mL kanamycin.

### 3D Collagen Gel Matrix Assay

Overnight cultures of *S. aureus* were back-diluted 1:100 into fresh TSB and grown 2h to exponential phase. The culture was then further diluted 1:10,000 and 50μL (~2,500 cells) was pelleted at 20,000 × g. The bacterial pellet was resuspended in a 20μl solution of rat tail collagen (ThermoFisher Scientific, cat no. 1048301) and RPMI + L-glutamine (ThermoFisher Scientific, cat no. 11875093) with a final concentration of 3.7mg/mL collagen. The suspension was pipetted into the center of a well in a 96-well plate (Greiner, cat no. 655180) and placed at 37°C for 45 min to allow the collagen matrix to solidify. 48μL overlay media from a master mix consisting of 25μL 10% pooled human plasma reconstituted in PBS (phosphate-buffered saline), 15μL of 10mg/mL human fibrinogen (Millipore Corp, cat no. 341576) reconstituted in PBS, 4μL of 53μg/mL prothrombin (Invitrogen, cat no. RP-43087) reconstituted in 50% glycerol, and 8μL of RPMI + L-glutamine was added onto each matrix. The plate was placed at 37°C until usage in downstream assays. We used a humidified environment for all incubation steps. In this manuscript, the time of growth within the matrix is listed as the time post overlay media addition.

### Plasma Sources

Two different plasma sources were used in experiments due to plasma sourcing/supply chain issues. All experiments except the flow cytometry, quantitative portions of neutrophil experiments, and second batch of cytotoxicity assay were conducted using lyophilized pooled human citrated plasma from Millipore-Sigma (cat no. P9523) reconstituted in PBS (100mg powder per 1mL PBS). The aforementioned experiments were conducted using pooled human citrated plasma from Innovative Research Inc (cat no. IPLAWBNAC50ML). This plasma source was delivered frozen at −80°C, lyophilized for 48h, then reconstituted in PBS (100mg powder per 1mL PBS) and filtered using a 5μm filter. SACs grown in both sources were assessed via microscopy, ensuring similar morphology and growth kinetics.

### Microscopy

The 96-well plate containing matrices with overlay media was placed in the Zeiss Axio Observer 7 inverted fluorescent microscope. Images were captured with an Axiocam 702 mono camera (Zeiss) and processed using ZEN3.10 software. Using a 5X objective, the field of view was adjusted to the center of a well and then further adjusted along the z-plane to the midline of the matrix. For timelapse microscopy, the chamber was pre-warmed to 37°C, and image acquisition was automated to take images across all matrices of a given 96-well plate every 30 minutes.

### Microscopy Quantification

Only SACs in focus, fully in the plane of view of a given image, and not touching other SACs, were included in quantification. Fluorescent signal was quantified using Volocity software (Quorum Technologies Inc). Except for the fibrin immunofluorescence experiment, background signal from SAC-less regions of a given matrix were subtracted from all fluorescence quantification. For experiments where individual SACs were tracked overtime, background signal from the area immediately adjacent to SAC was subtracted from each SAC; for experiments where individual SACs were not tracked overtime (i.e. the population was tracked overtime) the background signal was taken from one or two SAC-less areas that were representative of overall background signal in the matrix.

### Fibrin Immunofluorescence

Gel matrices were grown containing either WT or Δ*coa* Δ*vwbp* *S. aureus*. Wells were fixed with 4% PFA for 30min at room temperature after 18h of growth. Matrices were washed with PBS then blocked with 2% BSA solution for 1hr at room temperature. Matrices were washed with PBS then incubated overnight at 4°C with 100μL of a 1:100 dilution of anti-fibrin primary antibody (Sigma-Aldrich, cat no. MABS2155) in 2% BSA. Matrices were washed with PBS then incubated at room temperature for 1hr with 100μL of a 1:500 dilution of secondary antibody (ThermoFisher Scientific, cat no. A21120) in 2% BSA. Matrices were washed with PBS, then 50μL PBS was added before imaging.

### RNA Isolation

Samples fixed in 4% PFA were processed for bulk RNA isolation. Fixation lasted for 30 min at 4°C for experiments validating matrix fluorescent protein expression, remaining experiments were either fixed for 30min at room temperature or 4°C overnight. For experiments validating matrix fluorescent protein expression, a 2mm biopsy pen was used to extract central contents of the gel matrices and placed in a 1.5mL microcentrifuge tube. Matrices were washed with PBS and centrifuged to isolate matrix contents, then resuspended in a PBS solution containing 1U collagenase enzyme. Matrices were incubated until visibly dissolved (enough to suspend into solution in subsequent steps), typically 30min at 37°C. Matrices were washed with PBS and centrifuged, then resuspended in 1mg/mL lysostaphin in 100mM Tris HCl (Millipore Sigma, cat no. L7386) and incubated at 37°C 30min. RNA isolation was performed using reagents from the TRIzol Max Bacterial RNA Isolation Kit (Invitrogen, cat no. 16096020) on the digested samples, followed by the Turbo DNA-free kit (Invitrogen, cat no. AM2238) to reduce gDNA contamination, according to manufacturer’s protocols.

For all other experiments, RNA from 4% PFA fixed samples was isolated using the RNeasy FFPE Kit (QIAGEN, cat no. 73504) according to manufacturer’s protocols. For gel matrices, mechanical disruption with sterile rotors and collagenase digestion were used to digest samples. This matrix digestion (or fixed planktonic samples) was then spun and resuspended and incubated in 1 mg/mL lysostaphin in 100mM Tris HCl no more than 30min at 37°C. RNaseOUT (Invitrogen, cat no. 10777019) was used during this digestion step in early iterations of this method. The RNeasy FFPE kit was then used to isolate RNA from the resulting lysate. The On-column DNase digestion kit (QIAGEN) was used according to manufacturer’s protocols. If residual gDNA was detected by RT-qPCR controls, the Turbo DNA-free kit was subsequently used according to manufacturer’s protocols.

### cDNA Synthesis, and RT-qPCR

To generate cDNA, the ProtoScript II First Strand cDNA Synthesis Kit was used (New England Biolabs, cat no. E6560S). Resulting cDNA was diluted in 80μL nuclease-free water and stored at −20°C. For the qPCR reactions, primers for *agrB*, *saeP*, *vraR, hlgA, sspA, lukB*, and 16s rRNA were used in concert with the PowerUp SYBR Green Master Mix (Applied Biosystems). Signal from the reaction was captured on a StepOnePlus System (Thermo Fischer Scientific). Relative quantification was assessed using ΔC_T_ values (16s C_T_ value subtracted from target C_T_ values of corresponding sample). All kits and reagents were used according to the manufacturers’ protocols.

### RNA Sequencing and Analysis

For RNA-seq, Genewiz (from Azenta Life Sciences) performed library preparation and paired end Illumina sequencing on total RNA samples. Downstream sequencing analysis and differential gene expression analysis was performed by the Davis Lab. The following software packages were used for each step: quality control was assessed using FASTQC, adaptor trimming using the bbduk, alignment was done using bowtie2, hit counts were tallied using featureCounts, and differential gene expression analysis was done via DESeq2 (58). The following reference genome was used for the analysis: ASM1708v1.

### Antibiotic Susceptibility

Overnight cultures of *S. aureus* strains were back-diluted 1:100 in fresh TSB and incubated 2h at 37°C with rotation for log phase samples. Cultures were then diluted 1:10 in TSB in technical replicates (~5 × 10^7^ CFU/mL). Technical replicates were given 50, 25 or 0 μg/mL vancomycin. Samples were incubated at 37°C with rotation for 5h then plated for viable counts. To assess antibiotic susceptibility in matrices, matrices were prepared and grown in technical replicate pairs for the indicated amounts of time. One matrix in each technical replicate pairing was treated with 50μg/mL vancomycin while the other was left untreated. Treatment lasted 5h, then matrices were digested with collagenase until the matrix was visibly dissolved, approximately 30 minutes at 37°C. Matrices were then plated for viable counts.

### BODIPY-Vancomycin Staining & Flow Cytometry

Matrices were grown for 8.5h, washed 1x with PBS, then incubated +/− 4μg/mL BODIPY-vancomycin conjugate (ThermoFisher Scientific, cat no. V34850). Matrices were incubated for 10 minutes at 37°C, washed, then fixed with 4% PFA. Matrices were mechanically disrupted with sterile rotors and digested with collagenase for approximately 30 minutes at 37°C. Digested matrices were then placed in FACS tubes and run through the BD FACSymphony A3 flow cytometer.

### Reseeding Assay

Matrices were grown for 19.5h, then digested with collagenase for 15 minutes at 37°C. The digestion was pelleted and resuspended in 1mL TSB, then diluted 1:1000, and 50μL of the dilution was spun down and incorporated into new gel matrix (~2,500 CFUs). Fresh overlay media was added immediately upon matrix seeding. Matrices were grown another 19.5h then digested with collagenase again. Aliquots were plated for viable counts, while the rest was diluted 1:10 in fresh TSB and incubated at 37°C with rotation. Absorbance (600nm) measurements were taken at the indicated timepoints from these planktonic cultures.

### Agarose Pads

Overnight cultures of *P*_*saeP*_::*gfp S. aureus* were split into technical replicate pairs and given 50μg/mL vancomycin or left untreated and placed at 37°C with rotation. 20μL of solution was sampled and fixed in 4% PFA per tube 0, 2, and 5h after antibiotic addition. Agarose pads were prepared to immobilize bacteria and assess bacterial fluorescence by microscopy. 25μL of 1% agarose dissolved in PBS was pipetted onto a standard microscope slide, covered by a coverslip, and left to solidify at room temperature for 30 minutes. Once the agarose solidified, the coverslip was removed and ~8μL of fixed bacteria were added. The pipetted bacteria were then covered by a fresh coverslip and imaged at 63X magnification using the fluorescent microscopy setup described above (59,60).

### PMN Isolation & Gel Matrix Incorporation

Buffy coats were obtained from the American Red Cross (ARCBS IRB: 2020-017). Samples were spun at 800 × g for 10min to separate red blood cells (RBCs). The immune cell-containing supernatant was then placed on a Mono-Poly Resolving Medium (MP Biomedicals, cat no. 1698049) in a 1:1 ratio and centrifuged for 45min at 800 × g. The granulocyte layer was then removed and processed to remove remaining RBCs. 1mL Red Blood Cell Lysis Buffer (Roche, cat no. 77443200) was mixed with 500μL granulocytes for 10 minutes at room temperature. Contents of this mixture were spun at 500 × g for 5 min, and the resulting pellet was suspended in another 1mL of Red Blood Cell Lysis Buffer. After another 500 × g for 3 minute spin, purified neutrophils were resuspended in media and incorporated into the appropriate gel matrices (~100,000 neutrophils). Neutrophils were added either 40 minutes (representative images) or 2h (quantitative analysis) after seeding. For each isolation, the purity of immune cell preparations was confirmed by flow cytometry. Neutrophils were identified as CD66b^+^CD49d^−^ using a CD66b monoclonal antibody (G10F5, APC; Thermo Fisher Scientific, cat no. 17-0666-42) and a CD49d monoclonal antibody (9F10, PE; Thermo Fisher Scientific, cat no. 12-0499-42).

### LDH Cytotoxicity Assay

WT, Δ*coa* Δ*vwbp*, and Δ*aur* SACs were grown in gel matrices for 19.5h using 150μL of RPMI + L-glut without phenol red as the RPMI source. Matrices were isolated from wells, placed in microcentrifuge tubes, and spun at 20,000×g. The supernatant was removed, placed in separate tubes, and stored at 4°C. Once PMNs were isolated, we seeded relevant wells of a V-bottom plate (Greiner, cat no. 651180) with ~500,000 PMNs in 140μL matrix supernatant. As a positive control, PMNs were resuspended in 140μL matrix supernatant with Triton X-100. As a negative control, PMNs were resuspended in RPMI + L-glut without phenol red. To account for media background signal, RPMI + L-glut without phenol red and matrix supernatant were placed in wells without PMNs. The V-bottom plate was incubated for 30 minutes at 37°C with 5% CO_2_, then spun at 400xg for 5 min. The top 100 μL of each well was transferred to a flat bottom 96-well plate and 100 μL of LDH reagent (Cell Signaling Technology, cat no. #37291) was mixed in each well, followed by incubation for 30min at 37°C, protected from light, with shaking. A_490nm_ was quantified as a readout for LDH release.

## Supplementary Material

Supplemental Datasets

**Supplemental Dataset Table:** all data points plotted in the main manuscript and Supplemental Figures. Each tab represents one Figure.

**Supplemental Fig 1. Primary neutrophils impact SAC biology in the gel matrix system. A)** Representative images of SACs grown with or without 100,000 primary human neutrophils (PMNs), imaged 20h after overlay media addition. PMNs were added after 40 min of SAC growth. **B-D)** *P*_*saeP*_*::gfp* SACs were grown in gel matrix system (3 biological replicates). For each biological replicate, one technical replicate received 100,000 PMNs after 2h of SAC growth while another used none. Endpoint analysis comparing CFUs per matrix (B), average SAC area (μm^2^) (C) and GFP adjusted MFI signal (D) were conducted after 6h of SAC growth. Statistics: **B-C)** Welch’s t-test; **D)** Mann Whitney U test (depending on the normality of the groups being compared according to the Shapiro-Wilk test); ** P<0.01, *P<0.05, ns P>0.05.

**Supplemental Fig 2. *S. aureus* strain growth dynamics in planktonic culture vs. in gel matrix system. A)** Blank-subtracted A_600_ measurements of *S. aureus* strains overtime. Overnight cultures were back diluted 1:100 into TSB and grown on a 37°C rotor. **B)** Bacterial load (CFU/mL) was measured at a subset of timepoints from cultures setup in (A). **C)** Gel matrices were grown and visualized using timelapse microscopy. Total bacterial area (μm^2^) in the center of each matrix was assessed per strain per timepoint (3–5 biological replicates per strain).

**Supplemental Fig 3. *S. aureus* planktonic response to different dosages of vancomycin. A)** WT overnight cultures were diluted 1:100 in TSB for 2h on 37°C rotor. These cultures were further diluted 1:10 in TSB and treated with 0, 25, or 50μg/ml vancomycin for 5h on a 37°C rotor. **B)** *P*_*saeP*_*::gfp* overnight cultures were treated with 0 or 50μg/mL vancomycin, then fixed and imaged on 1% agarose pads at various timepoints. Statistics: **A)** one-way ANOVA with Sidak’s multiple-comparison correction; **B)** multiple unpaired t-tests with Holm-Sidak correction; * P<0.05; ns P>0.05.

**Supplemental Table 1. RNA-seq data of WT Planktonic Bacteria in Log or Stationary Phase +/− 25μg/mL Vancomycin (All Genes).** Comparisons are made between subsets of conditions within the sequencing data set (one comparison per tab). Each gene is represented by the gene_id listed in the reference genome, the canonical gene_name if present, a description of the effector function of the gene product, the log_2_FoldChange, Fold Change, and adjusted p-value. No filtering criteria was applied; all genes with reads are listed. Treatment started after 14.5h of growth and lasted 5h. 3 biological replicates per condition across 2 batches.

**Supplemental Table 2. RNA-seq data of WT and Δ*coa* Δ*vwbp* SACs +/− 50μg/mL Vancomycin (All Genes).** Comparisons are made between listed subsets of conditions within the sequencing data set (one comparison per tab). Each gene is represented by the gene_id listed in the reference genome, the canonical gene_name if present, a description of the effector function of the gene product, the log2FoldChange, Fold Change, and adjusted p-value. No filtering criteria was applied; all genes with reads are listed. Treatment started after 14.5h of growth and lasted 5h. 5 biological replicates per condition across 2 batches.

**Supplemental Table 3. Gene Ontology Data for Stationary Phase SAC RNA-seq Data.** Gene Ontology (GO) Biological Process (BP) enrichment was performed on genes differentially expressed from RNA-seq data using the topGO package. Enrichment was assessed separately for significantly up and down genes (adjusted p value < 0.05, |log_2_FoldChange| >1, and baseMean >75 reads) using the topGO package with the weight01 algorithm and Fisher’s exact test. The gene universe was restricted to genes detected in the RNA-seq dataset and annotated with at least one GO term. Shown are the top 20 enriched GO terms per analysis; listed genes represent those contributing to each enriched term. Weighted Fischer output is unadjusted.

## Figures and Tables

**Table 1.** RNA sequencing data of WT and Δ*coa* Δ*vwbp* SACs +/− 50μg/mL Vancomycin (Significant Genes). Comparisons are made between subsets of conditions within the sequencing data set. Each gene is represented by the gene_id listed in the reference genome, the canonical gene_name if it is present, a description of the effector function of the gene product, the log_2_FoldChange, Fold Change, and adjusted p-value for each gene. Cutoffs of log_2_FoldChange +/− 2.32, baseMean >75 reads, and adjusted p-value <0.05 were used as inclusion criteria for genes in each comparison. Treatment started after 14.5h of growth and lasted 5h. 5 biological replicates per condition across 2 batches.

WT Untreated vs. Δ*coa* Δ*vwbp* Untreated (Negative = Up in WT Untreated)					
gene_id	gene_name	Description	log2FoldCha	Fold Change	padj
USA300HOU_RS03030	vraX	C1q-binding complement inhibitor VraX	4.95327427	30.9801939	0.00667542
USA300HOU_RS10300	rlmD	23S rRNA (uracil(1939)-C(5))-methyltransferase RlmD	4.18104919	18.1393291	0.00014599
USA300HOU_RS13875	cwrA	cell wall inhibition responsive protein CwrA	4.04463012	16.5026993	0.00031003
USA300HOU_RS15345	USA300HOU_RS15345	hypothetical protein	3.90391089	14.9690513	3.26E-06
USA300HOU_RS13180	USA300HOU_RS13180	membrane protein	3.85029713	14.4229776	1.68E-05
USA300HOU_RS13095	hlgA	bi-component gamma-hemolysin HlgAB subunit A	3.84412632	14.3614182	2.90E-06
USA300HOU_RS10235	USA300HOU_RS10235	hypothetical protein	3.68552399	12.8662881	1.44E-05
USA300HOU_RS12570	USA300HOU_RS12570	hypothetical protein	3.59263233	12.0639658	4.93E-06
USA300HOU_RS13175	USA300HOU_RS13175	glycerate kinase	3.50808802	11.3773134	3.58E-05
USA300HOU_RS08780	USA300HOU_RS08780	DUF4930 family protein	3.47225798	11.0982322	2.29E-05
USA300HOU_RS13100	hlgC	bi-component gamma-hemolysin HlgCB subunit C	3.45887679	10.9957704	1.66E-07
USA300HOU_RS10225	vraS	sensor histidine kinase VraS	3.3302487	10.0578407	2.32E-05
USA300HOU_RS10230	liaF/vraT	cell wall-active antibiotics response protein VraT	3.30501462	9.88344927	3.51E-05
USA300HOU_RS13085	sbi	immunoglobulin-binding protein Sbi	3.23141479	9.39188528	1.66E-08
USA300HOU_RS08775	USA300HOU_RS08775	hypothetical protein	3.18827223	9.11518682	2.36E-05
USA300HOU_RS10220	vraR	response regulator transcription factor VraR	3.18278479	9.08058213	2.53E-05
USA300HOU_RS03025	USA300HOU_RS03025	hypothetical protein	3.14948257	8.87337274	2.29E-05
USA300HOU_RS03020	USA300HOU_RS03020	protein VraC	3.14096933	8.82116577	2.29E-05
USA300HOU_RS09835	USA300HOU_RS09835	peptidylprolyl isomerase PrsA	3.01828795	8.10205541	0.00031921
USA300HOU_RS03440	USA300HOU_RS03440	M50 family metallopeptidase	2.96873911	7.82851742	0.00012937
USA300HOU_RS05775	ecb	complement convertase inhibitor Ecb	2.95180989	7.73719099	1.87E-06
USA300HOU_RS10295	USA300HOU_RS10295	DUF3267 domain-containing protein	2.94749113	7.7140641	0.00020614
USA300HOU_RS05800	scb	complement inhibitor SCIN-B	2.91999255	7.56842208	4.52E-07
USA300HOU_RS05795	efb	complement convertase inhibitor Efb	2.89989955	7.46374421	1.52E-06
USA300HOU_RS14655	vraE	peptide resistance ABC transporter permease subunit V	2.83420386	7.13149159	0.00032548
USA300HOU_RS14650	vraD	peptide resistance ABC transporter ATP-binding subunit	2.72935958	6.63161192	0.00026713
USA300HOU_RS14590	USA300HOU_RS14590	SMP-30/gluconolactonase/LRE family protein	2.60752044	6.09455312	0.00063198
USA300HOU_RS07275	msrB	peptide-methionine (R)-S-oxide reductase MsrB	2.59733739	6.05168706	0.00045522
USA300HOU_RS13550	fnbB	fibronectin-binding protein FnbB	2.58913246	6.01736744	1.38E-05
USA300HOU_RS10170	sgtB	monofunctional peptidoglycan glycosyltransferase SgtB	2.52267585	5.74646942	0.00055685
USA300HOU_RS07280	msrA	peptide-methionine (S)-S-oxide reductase MsrA	2.44258243	5.43613932	0.00049489
USA300HOU_RS03015	USA300HOU_RS03015	thiolase family protein	2.43045558	5.39063633	2.84E-05
USA300HOU_RS07270	USA300HOU_RS07270	PTS sugar transporter subunit IIA	2.40162495	5.28397979	0.00069549
USA300HOU_RS05215	sspA	Glu-specific serine endopeptidase SspA	−5.1545301	0.02807578	5.12E-16
USA300HOU_RS05210	sspB	cysteine protease staphopain B	−4.7882506	0.03619036	5.12E-16
USA300HOU_RS05205	sspC	staphostatin B	−4.4848613	0.04466036	1.63E-14
USA300HOU_RS15710	USA300HOU_RS15710	phenol-soluble modulin PSM-alpha-4	−4.3604083	0.04868401	2.02E-14
USA300HOU_RS15050	USA300HOU_RS15050	phenol-soluble modulin PSM-alpha-3	−4.3545141	0.04888331	2.49E-15
USA300HOU_RS14335	aur	zinc metalloproteinase aureolysin	−4.338491	0.04942926	7.66E-12
USA300HOU_RS15715	USA300HOU_RS15715	phenol-soluble modulin PSM-alpha-2	−3.8256409	0.07052894	8.84E-08
USA300HOU_RS02035	USA300HOU_RS02035	hypothetical protein	−3.5440668	0.08572936	2.42E-10
USA300HOU_RS16075	USA300HOU_RS16075	phosphatidylinositol-specific phospholipase C (PI-PLC)	−2.7077481	0.15306877	5.81E-05
WT Treated vs. Δ*coa* Δ*vwbp* Treated (Negative = Up in WT Treated)					
gene_id	gene_name	Description	log2FoldCha	Fold Change	padj
USA300HOU_RS05215	sspA	Glu-specific serine endopeptidase SspA	−5.6291511	0.0202049	1.61E-27
USA300HOU_RS05210	sspB	cysteine protease staphopain B	−5.0578731	0.03002123	2.20E-25
USA300HOU_RS05205	sspC	staphostatin B	−4.5317812	0.04323126	2.32E-22
USA300HOU_RS14335	aur	zinc metalloproteinase aureolysin	−4.4365248	0.04618202	1.25E-15
USA300HOU_RS00935	USA300HOU_RS00935	NAD-dependent formate dehydrogenase	−3.5370667	0.08614634	1.45E-07
USA300HOU_RS01175	coa	coagulase	−2.6453298	0.15983666	9.82E-06
USA300HOU_RS12385	USA300HOU_RS12385	urease subunit beta	−2.3522034	0.19584669	5.00E-11
WT Untreated vs. WT Treated (Negative = Up in WT Untreated)					
gene_id	gene_name	Description	log2FoldCha	Fold Change	padj
USA300HOU_RS03030	vraX	C1q-binding complement inhibitor VraX	3.85292106	14.4492336	1.83E-50
USA300HOU_RS13095	hlgA	bi-component gamma-hemolysin HlgAB subunit A	3.34485458	10.1601837	8.59E-15
USA300HOU_RS13180	USA300HOU_RS13180	membrane protein	3.34477205	10.1596025	2.27E-36
USA300HOU_RS10300	rlmD	23S rRNA (uracil(1939)-C(5))-methyltransferase RlmD	3.32117607	9.99478871	4.31E-46
USA300HOU_RS13875	cwrA	cell wall inhibition responsive protein CwrA	3.31758349	9.96993076	7.10E-35
USA300HOU_RS10235	USA300HOU_RS10235	hypothetical protein	3.044509	8.25065698	9.32E-33
USA300HOU_RS12570	USA300HOU_RS12570	hypothetical protein	2.95561838	7.75764295	1.26E-39
USA300HOU_RS13175	USA300HOU_RS13175	glycerate kinase	2.92558484	7.59781634	1.06E-34
USA300HOU_RS15345	USA300HOU_RS15345	hypothetical protein	2.86837613	7.30242751	1.29E-29
USA300HOU_RS13100	hlgC	bi-component gamma-hemolysin HlgCB subunit C	2.74264381	6.69295731	3.35E-12
USA300HOU_RS08780	USA300HOU_RS08780	DUF4930 family protein	2.63853466	6.2269887	2.28E-27
USA300HOU_RS05800	scb	complement inhibitor SCIN-B	2.4915068	5.62364998	1.49E-10
USA300HOU_RS05795	efb	complement convertase inhibitor Efb	2.48987386	5.61728833	1.03E-09
USA300HOU_RS14650	vraD	peptide resistance ABC transporter ATP-binding subunit	2.48828166	5.61109234	3.74E-24
USA300HOU_RS10230	liaF/vraT	cell wall-active antibiotics response protein VraT	2.45510321	5.48352349	6.26E-35
USA300HOU_RS14655	vraE	peptide resistance ABC transporter permease subunit V	2.42205823	5.35935073	6.17E-23
USA300HOU_RS09835	prsA	peptidylprolyl isomerase PrsA	2.41374383	5.32855305	1.64E-28
USA300HOU_RS12745	tcaA	glycopeptide resistance protein TcaA	2.37896595	5.20163781	3.65E-36
USA300HOU_RS10225	vraS	sensor histidine kinase VraS	2.37175367	5.17569883	1.81E-32
USA300HOU_RS14590	USA300HOU_RS14590	SMP-30/gluconolactonase/LRE family protein	2.33878061	5.05874884	9.93E-28
USA300HOU_RS08775	USA300HOU_RS08775	hypothetical protein	2.32265347	5.00251459	2.57E-20
